# Spatial-temporal evolution of the allometric relationship between urban economic and health resources in the Yangtze River Delta urban agglomeration

**DOI:** 10.1371/journal.pone.0314315

**Published:** 2025-01-24

**Authors:** Deng Jing, Qianwen Song, Huan Liu, Zicheng Jiang, Xingxing He, Chengzhi Ge, Dexun Li

**Affiliations:** 1 School of Pharmaceutical Economics and Management, Anhui University of Traditional Chinese Medicine, Hefei, Anhui, China; 2 Key Laboratory of Philosophy and Social Science for Data Science and Innovative Development of Traditional Chinese Medicine, Hefei, Anhui, China; 3 Office of Maternal and Child Health Hospital, Shuangliu District, Chengdu, Sichuan, China; 4 Ya’an Center for Disease Prevention and Control, Yuancheng District, Ya’an, Sichuan, China; 5 The Affiliated Jiangyin Hospital of Southeast University Medical College, Jiangyin, China; 6 School of Management, Xihua University, Chengdu, Sichuan, China; Tongji University School of Economics and Management, CHINA

## Abstract

The evolution of the spatiotemporal relationship between urban economic growth and health resources within the Yangtze River Delta urban agglomeration provides an important context for understanding the regional development dynamics in China. Previous studies focused on equity in health-resource allocation and service efficiency, often overlooking the allometric growth relationships between health resources and economic variables. This study employs an allometric growth model to elucidate the changing interactions between the number of medical beds, doctors, and urban economic indicators in the Yangtze River Delta region from 2009 to 2022. Employing Zipf’s law and allometric growth modeling, this study analyzed growth trends and revealed significant differences in resource allocation and size changes over time. The main findings suggest that, although resource centralization is a general trend, differences persist, especially in less economically developed regions. This study innovatively introduces an allometric growth model that offers a new perspective on understanding the mechanisms of regional health-resource growth and underscores the significant influence of economic factors on health-resource allocation. This study significantly contributes to the sustainability of urban health systems and provides theoretical support for policy formulations aimed at optimizing the allocation of health resources and strengthening regional economic strategies in the Yangtze River Delta region.

## Introduction

Healthcare beds and doctor resources constitute the essential components of China’s healthcare services and are crucial indicators of urban healthcare capacity. Over the past three decades, China has witnessed significant economic growth and transition towards a market-based economy. According to the China Bureau of Statistics, China’s GDP expanded from 367.8 billion yuan in 1978 to 121 trillion yuan by 2022. Although this paradigm shift has bolstered the expansion of China’s total healthcare resources, it has significantly impacted the public health service system [1], exacerbating regional disparities in healthcare resources [2], urban-rural divides [3], and inefficiencies in health services [4]. Among these issues, the uneven distribution of medical and health resources at the regional level is particularly pronounced, with a continuing trend towards increasing intra-regional disparities [5]. Consequently, the uneven distribution of healthcare resources and the need to reduce intra-regional disparities have become pressing concerns for both Chinese society and governments [6].

Since 2009, China has initiated reforms in its health system and has progressively increased health investments in the central and western regions, aiming to rectify the uneven distribution of health resources through governmental macro policies. While these macro policies have yielded some success, they have also engendered new issues owing to governmental shortcomings and inadequate comprehension of the relationship between the economy and health resources. This has resulted in public health services lagging behind economic development and failing to meet the health needs of the population [7,8]. Conversely, the absence of rational intercity health-resource planning has led to poor cooperation across urban health systems, redundant health facilities, and diminished overall utilization efficiency. Healthcare development is intrinsically linked to the urban economy and its progress directly influences the future growth potential of the urban economy. Consequently, elucidating the intrinsic connection between health resources and the urban economy is crucial for achieving high-quality urban economic development and optimizing the rational distribution of health resources across cities [9].

Presently, the Yangtze River Delta (YRD) is one of China’s regions characterized by vigorous economic development, high population density, and the early initiation of regional integration. However, owing to marked disparities in initial economic levels, geographic locations, policy support, and natural endowments among cities, rapid economic development has intensified intra-regional differentiation and increasingly unbalanced development across cities, particularly in healthcare services, where the issue of imbalance has grown increasingly pronounced within city clusters. Overlooking the intrinsic connection between economic development and health-resource allocation in urban areas poses a latent risk to sustained and robust development in the YRD region. Considering these factors, we selected the YRD region as our research subject to examine the inherent link between its urban economy and health resources (numbers of beds and doctors). Accordingly, the study’s objectives are multifaceted. the first is to investigate the characteristics and evolutionary trends of the health-resource scale system in the YRD region. This analysis scrutinizes the internal dynamics shaping these trends. Second, this study explores the interactions between health resources and economic development in the YRD region in light of China’s recent healthcare reforms. Third, we investigate the impact of YRD integration planning on health-resource development, offering insights into regional policy effects [10].

The study’s research objectives are diverse. the first is to explore the characteristics and evolutionary trends of the health-resource scale system in the YRD region. This entails analyzing the underlying dynamics that shape these trends. Second, this study examines the interplay between health resources and economic development within the YRD in the wake of China’s recent healthcare reforms. This analysis employed an allometric growth model to assess the relationship between health-resource indicators (beds/doctors) and economic factors. Third, the impact of YRD integration planning on health-resource development, offering insights into regional policy effects are explored [11]. Overall, we aimed to deepen the understanding of the nexus between health resources and economic factors within urban agglomerations, thereby providing theoretical and practical insights into enhancing the integrated development of health and economic systems in the YRD [12].

### The main work and contributions of this paper

**Fig 1:**
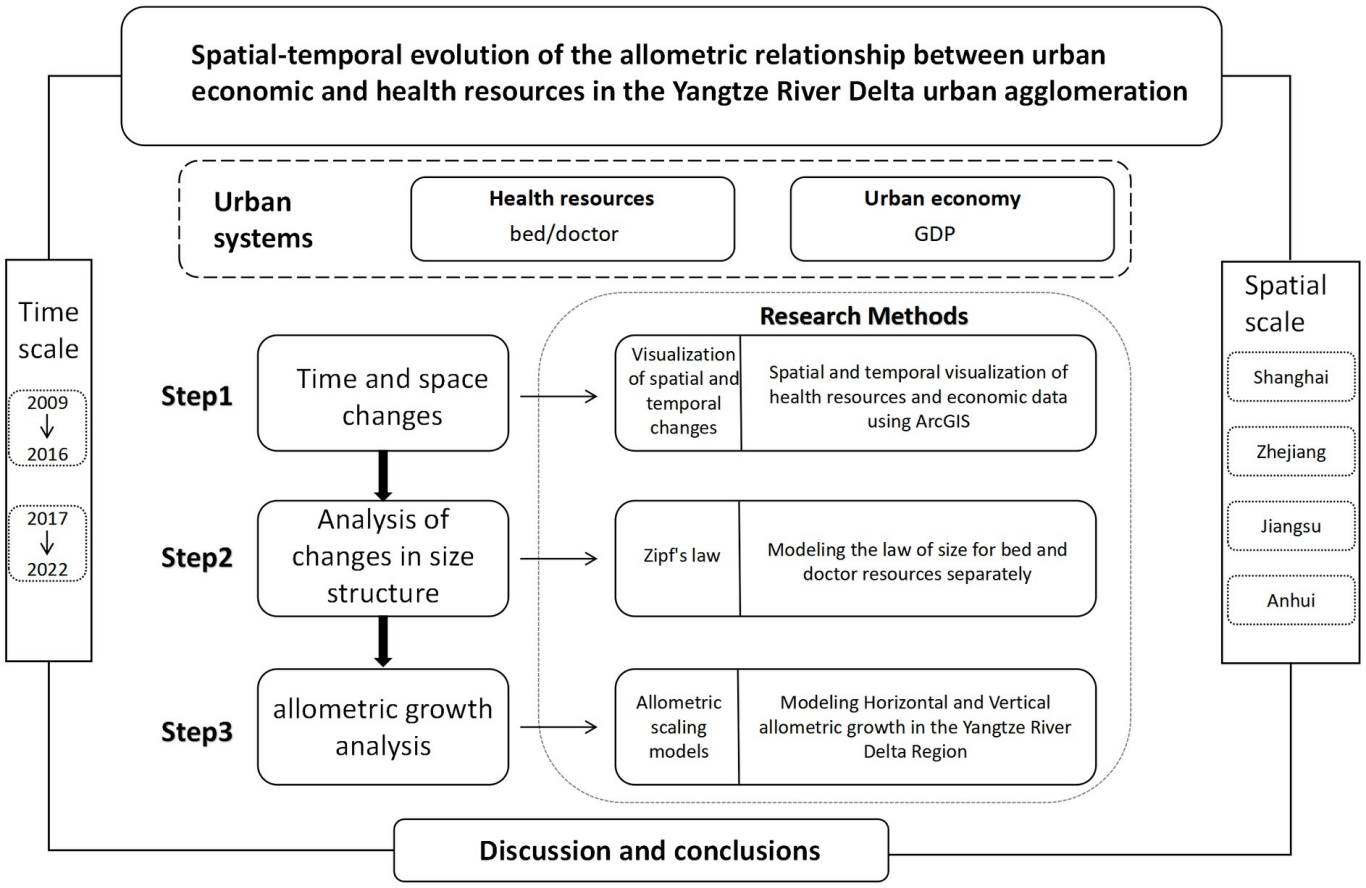
The research processes for this paper.

Fig 1 illustrates our analysis of the changes in the size structure of urban health resources (number of beds/doctors) in the YRD region as well as the changes in relative growth rates of the urban economy and health resources using the law of urban regularity and the allometric growth model, respectively. Initially, we employed the ArcGIS tool to visualize and analyze the overall growth and spatiotemporal historical changes in economic and health resources in the YRD region. Subsequently, we utilized the city-scale method to calculate the scale change index of various health resources in YRD urban health and analyzed their development trends. Finally, by employing the allometric growth model, we calculated both horizontal and vertical allometric growth models for the YRD region and visualized their spatiotemporal change characteristics.

This study contributes to the literature in three ways. First, it analyzed the structural allocation of health resources in the YRD region by employing the law of city size to elucidate changes in the scale of health resource development. Previous studies have primarily focused on health-resource allocation equity [11–13] and service efficiency [14,15]; however, analyses of the structural dynamics of health-resource scales remain insufficient. Second, by employing an allometric growth model, this study explored the relationship between health-resource indicators (beds and physician resources) and urban economic growth in the YRD region. Although some studies [16,17] have begun to investigate this relationship, comprehensive analyses that integrate health resources with economic variables remain scarce. Finally, based on the Integrated Development Plan for the YRD Region [18], this study evaluates the urban health system within the framework of city clusters and proposes strategies to enhance the rational allocation of health resources across administrative regions.

The remainder of this paper is organized as follows: The Literature review section reviews the literature. The Methods and materials section details the study area, data sources, and research methodology. The computational results are presented in the Results section. The Discussion section discusses the findings, policy recommendations, potential limitations, and future perspectives. Finally, the Conclusions section outlines the conclusions and potential future research directions derived from this study.

## Literature review

The relationship between health resources and economic development remains a pivotal research topic. Scholars largely agree on the interactive relationship between these two dimensions, wherein economic development not only facilitates the augmentation of health resources but also, the enhancement of health resources supports economic growth through various mechanisms [19–21]. Moreover, the application of allometric growth in this domain has been increasingly acknowledged.

### Study on the interaction between health resources and economic factors

Regarding the scope of research, economic development exerts a promotional effect on health services. For example, Zeng et al. employed regression analysis to explore the impact of economic agglomeration on urban public health and its mechanisms and found that economic agglomeration significantly promotes urban public health [22]. Tang utilized a coupled coordination model to determine whether health resources were highly correlated with the level of economic development and the impact of health services in supporting economic development. Using a panel co-integration model, Wang et al. [23] found that health equalization promotes regional economic growth. Reeves et al. [24] discovered that health expenditure significantly improves the quality of economic growth. Guo TJ et al. found that increasing health expenditures can effectively reduce the incidence of poverty and promote economic growth [25]; from the perspective of research methodology, treating urban economic factors and health resources as two systems of equal importance, explore the development relationship between economic factors and health resources, and assess the coordinated development status between the two by employing the coupled degree of coordination model, grey correlation analysis, and other methods [22,26]. Analyze the interrelationships between economic factors and health resources, as well as the influencing factors, employing methods such as the spatial convergence model, Markov chain, and spatial measurement models like geo-detector, and propose suggestions and initiatives for coordinated development [27–29]. Or use the Gini coefficient, the coefficient of variation method, the Terrell index, and other methods of health-resource allocation of economic fairness for research, with a view to narrowing the regional differences in health resources [30–32]. In addition, as the research on the relationship between health resources and economic development from the national, regional, provincial, and regional levels continues to explore the relationship between the level of health development and economic development more deeply, it has been shown that the health development level is higher than that of economic growth [33–35].

### Allometric scaling and urban health resources

The allometric growth model, originating from biology, is used to explain the scaling relationships between the size of an organ and the whole body or between two different organs during an organism’s growth [36]. Allometric growth has been increasingly applied in urban research and has emerged as a key principle linking the spatial distribution, hierarchical structure, and dynamic evolution of the two systems within a city. This model can also link the spatial distribution and dynamic evolution of two systems within a city [37]. Scholars have redefined this model to represent the constant proportionality between the local relative growth rate of a system and that of the entire system or its components [38]. In urban research, the primary focus is on the relationship between the growth of various elements in urban development and the expansion of the urban scale [39], including the dynamics between urban land and population, urban economy and land, and health resources and population size [40–43]. Specifically, using an allometric growth model, XU SG et al. [16] demonstrated that health resources in Chinese county-level cities are generally less developed than those in larger cities. Han et al. identified an allometric scaling relationship between the density of healthcare facilities and the population density in China [17].

Although substantial progress has been made in understanding the interplay between health resources and economic development, several shortcomings remain. First, although methods such as the entropy method have been employed to analyze the relationship between health resources and economic levels, there remains a lack of attention to the distinct characteristics of various elements within health resources and economic development. Second, although numerous studies have quantified the relationship between regional economies and health resources using diverse mathematical models to establish causality and effect processes, the focus on the relative development rates and growth dynamics of these variables remains insufficient. Third, the analysis of allometric growth in urban health-resource development remains underdeveloped, with existing research predominantly focusing on the correlation between urban population size and medical resources rather than the interplay with urban economic factors. Finally, while many studies have used panel data at the provincial or national level, the spatial significance of urban agglomerations as critical functional areas has been overlooked.

In light of the identified research gaps (Table 1), this study underscores the need to delve deeper into the nuanced aspects of health-resource distribution and economic interactions, with a particular focus on dynamic growth rates and spatial dimensions within urban agglomerations [44]. Addressing these issues is crucial not only for enriching the theoretical framework, but also for enhancing the practical knowledge essential for informed policymaking in urban health and economic planning. Consequently, this study explores the potential allometric growth relationship between the urban economy and health systems. Specifically, it aims to analyze this relationship in the YRD region of China, an area well suited for such an investigation, given its complex economic and health dynamics.

**Table 1:** Summary of research methods and focus in literature.

Research Method	Research Subject	Time Scope	References
Spatial Durbin Model	Digital Economy and Public Health	2010–2022	[19]
ARDL and Kalman Filter Modeling	Health Expenditure and Economic Growth	1975–2013	[20]
Toda-Yamamoto Causality Test	Health Expenditure and Economic Growth	2000–2019	[21]
GS2SLS	Economic Agglomeration and Resident Health	2003–2020	[22]
Generalized Additive Model	Government Health Expenditure and Economic Growth	1996–2017	[23]
Fixed Effects Models	Government Health Expenditure and Economic Growth	1995–2010	[24]
Sample Selection Model	Public Medical Services and Income of Residents	2016	[25]
Grey Relational Analysis, Coupling Coordination Models	Health Human Capital Investment and Regional Economy	2009–2013	[26]
Coupling Coordination Models and β-Convergence Models	Health Service Supply and Regional Economy	2009–2021	[27]
Markov Model	Medical Tourism Industry and Regional Economy	2013–2017	[28]
Coupling Coordination Model, Tobit Model	Traditional Chinese Medicine Medical Services and Regional Economy	2015–2021	[29]
Gini Coefficient, Theil Index	Public Medical and Health Resources	2016–2020	[30]
Theil Index, System GMM Model	Government Health Expenditure and Economic Growth	2015–2020	[31]
Entropic Method, Coefficient of Variation	Basic Public Health Services	2010–2020	[32]
SIMOE, MIMOE	Health Resources Allocation Efficiencies	2020	[33]
Gini Coefficient, Theil Index	Health Resources Allocation	2014–2018	[34]
Gini Coefficient, DEA	Maternal and Child Health Resources Allocation	2017	[35]
Allometric Scaling Model	Urban Economy and Carbon Emissions	2000–2017	[40]
Allometric Scaling Model	Material Infrastructure and Urban Area	1800–2000	[41]
Allometric Scaling Model	Urban Population and Income of Residents	2010–2011	[42]
Allometric Scaling Model	Urban Area and Urban Population	1949–1981	[43]
Allometric Scaling Model	Health Resources and Urban Population	2000–2015	[16]
Allometric Scaling Model	Medical Services and Population Density	2008–2014	[17]

Furthermore, recognizing that much of the existing research relies on data from 2016, which have diminished relevance post-COVID-19, this study aims to provide updated insights. It utilizes data from 41 cities in the YRD and employs both Zipf’s law and an allometric growth model. This study used hospital bed availability to represent physical health resources, and the number of doctors to measure human health resources. Separate growth models were constructed for the city economy, hospital beds, and medical staff. Analyses from both scale and allometric perspectives explore changes in the structural relationships and growth dynamics between health resources and the urban economy from 2009 to 2022. This comprehensive analysis aims to deepen our understanding of the interrelationships between health resources, specifically hospital beds, medical staff, and urban economic subsystems at the city cluster level. This study aims to establish a theoretical foundation and practical guidelines for enhancing the quality of health resources and economic development within the cities of the YRD region.

## Methods and materials

### Study area

**Fig 2:**
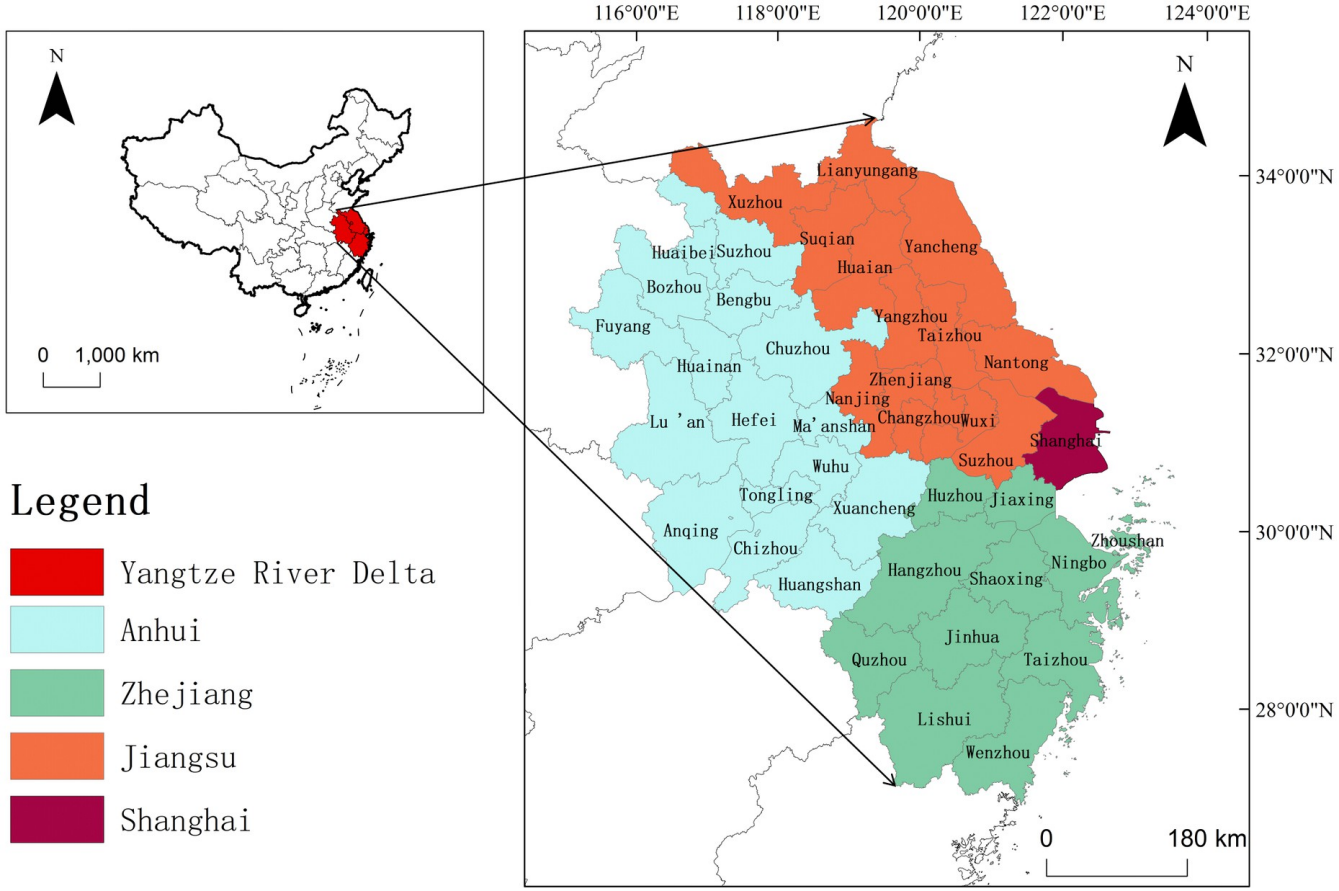
Overview of the study area.

According to the Outline of the Integrated Development Plan for the YRD, the region encompasses Shanghai, Zhejiang, Jiangsu, and Anhui provinces, comprising 41 cities (refer to Fig 2). The YRD is one of China’s largest urban agglomerations and exerts a substantial impact on the national economy by generating approximately one-quarter of China’s total economic output from less than 4% of the country’s land area, underscoring its central role in the national economic framework. The YRD is equipped with numerous high-level medical institutions and a wealth of health resources, which not only satisfy the basic medical demands of the population, but also significantly enhance the quality of public health services and advance healthcare system reforms. However, the rapid pace of economic growth has increasingly highlighted a critical misalignment between the healthcare infrastructure and economic expansion, presenting challenges to the region’s aspiration for high-quality and sustainable development.

### Data sources

This study focuses on health indicators such as the number of hospitals and primary-level hospitals, along with the number of doctors, including both practicing and assistant doctors, and the number of hospital beds. This study considered the total GDP of 41 cities in the YRD region as an economic indicator. Raw data spanning 2009–2022 were extracted from the China Urban Statistical Yearbook for each corresponding year.

### Research methodology

#### Zipf’s law

The city-size ranking law posits that the size hierarchy of cities exhibits recursive structural characteristics and analyzes them to reflect the discrete degree of city-size distribution in a country or region. Moreover, Zipf’s law requires less data than methods such as the Lorenz curve, Primacy, and the Prévost index and demonstrates varying degrees of fit to real situations. Additionally, empirical studies testing Zipf’s law on samples of cities across various countries have shown that it can effectively fit city size distribution to a certain extent. Consequently, this study applies Zipf’s law to analyze the characteristics and evolutionary trends of the bed/doctor-size system in the YRD region and explore the rationality of its distribution [45–47]. Its underlying formula:

$$P_{i}=P_{1}R_{i}^{\left(-q\right)}.$$ (1)

Taking the logarithms of both sides of Eq (1), the following expression is obtained:

$$LnP_{i}=LnP_{1}-qLnR_{i}.$$ (2)

In these equations, *P*_*i*_ represents the scale of beds/doctors for the *ith* city, *P*_1_ is the scale number for the first city, *R*_*i*_ denotes the city rank, and *q* is Zipf’s law coefficient. When *q* = 1, this corresponds to the standard "Zipf’s law" distribution, reflecting the optimal distribution of the natural state of the city’s beds/doctors. A *q* > 1 value greater than 1 signifies a Pareto distribution, indicating concentration, whereas a *q* < 1 value less than 1 suggests a normal distribution, indicating balance [48]. Additionally, when cities are sorted by size and regressed, autocorrelation among error terms can lead to estimation errors in *q* [49]. Research has demonstrated that converting the city rank order’s logarithm to (*R*−0.5) can effectively minimize this estimation error [50].

#### Allometric scaling models

Allometric growth, originating from biology, explores the geometric relationship between a part and an entire organism. This concept was later adopted in urban research to examine the proportionality of growth rates between systems in cities, often revealing a nonlinear relationship. Given the close relationship between a city’s economic level and health-resource development, economic growth supports the requisite material conditions for health resources, which can promote economic expansion. Conversely, the profit-seeking nature of health-resource investment can lead to a misalignment between resource allocation and economic development, causing asynchronous growth [51]. Additionally, because health resources and the urban economy represent different dimensions of factor variables, it is essential to apply an allometric growth model to assess the relative development rates and patterns of health resources. Allometric growth can be categorized into vertical and horizontal relationships. The formula is:

$$A=kG_{i}^{b}.$$ (3)

Taking the logarithms of both sides of Eq (3), the following expression is obtained:

$$LnA=k+bLnG_{i}.$$ (4)

Where *A* represents the number of beds or doctors in each city, *G*_*i*_ denotes the economic development level of city *i*, *b* is the scaling index, and *k* is a constant term: Existing studies [52,53] have shown that the scaling index *b* ranges from 2/3 to 1, with 0.85 commonly used as the threshold. Specifically, when *b* = 0.85, the urban economy and health-resource scales grow at an equal rate. If *b* > 0.85, termed positive allometric growth, the health-resource scale grows faster than the economic scale. Conversely, when *b* < 0.85, known as negative allometric growth, the health-resource scale grows slower than the economic scale. Integrating existing research with this study’s findings, the allometric relationships are further categorized as detailed in Table 2 [44].

**Table 2:** Classification of the allometric relationship between health resources and urban economy.

Type of allometric scaling	Allometric level	Standard	Characterization
Positive allometric scaling	Positive 3	*b* ≥ 2	Health resources are growing much faster than the urban economy
	Positive2	2 > *b* ≥ 1	Health resources are growing faster than the urban economy
	Positive1	1 > *b* ≥ 0.85	Health resources are growing slightly faster than the urban economy
Negative allometric scaling	Negative1	0.85 > *b* ≥ 0.5	Health resources are growing lower than the urban economy
	Negative2	0.5 >*b* ≥ 0	Health resources are growing much lower than the urban economy
	Negative3	0 > *b*	Decrease in either or both health resources and urban economy

Notes: *b* is the allometric scaling index.

## Results

### Spatial and temporal distribution of economic and health resources in the YRD region

The years 2009 and 2022 were selected as representative years, and ArcGIS software was used to map the spatial evolution of the urban economy, beds, and physician resources in the YRD region. Additionally, we standardized the data in ArcGIS using the ESRI percent total normalization method and subsequently classified them into five categories using the natural breakpoint method: leading, good, intermediate, low, and lagging.

Between 2009 and 2022, Fig 3 illustrates a significant upward trend in the economic output, number of beds, and number of doctors in the YRD region. Specifically, the total economic output increased from 8253.5 billion yuan in 2009 to 29,107.49 billion yuan in 2022, with a distinct pattern of higher economic activity in the east, lower activity in the west, and a concentration in central cities. During this period, the number of beds increased from 647,753 to 1,277,741 and the number of doctors increased from 351,070 to 796,879. Both resources exhibited similar spatial distributions, with higher concentrations in the eastern and southern parts than in the west and north, and a notable concentration in the central urban areas. Notably, the southern part of Anhui and western part of Zhejiang exhibited slower development in terms of both economic and healthcare resources. Overall, the development of healthcare resources in YRD cities mirrors economic trends, demonstrating a significant trend towards centralization. Despite this trend, healthcare resources are relatively balanced across the regions.

**Fig 3:**
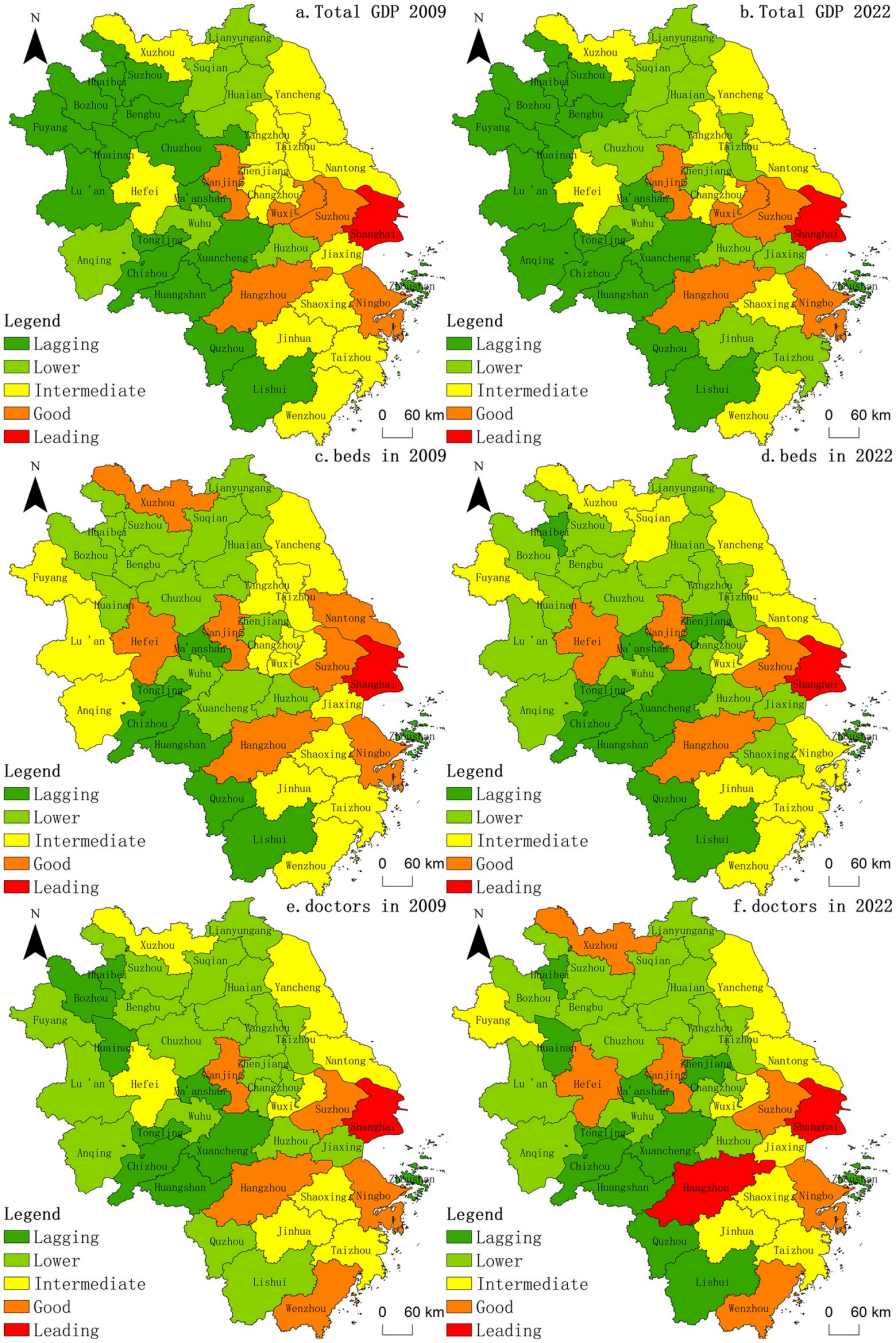
Spatial and temporal distribution of economic and health resources in YRD cities. Notes: Based on the standard map GS (2019)1822 of the standard map service website of the Ministry of Natural Resources, the map boundary was not modified during ArcGIS production.

### Zipf’s law regression analysis of the size of urban health resources

**Fig 4:**
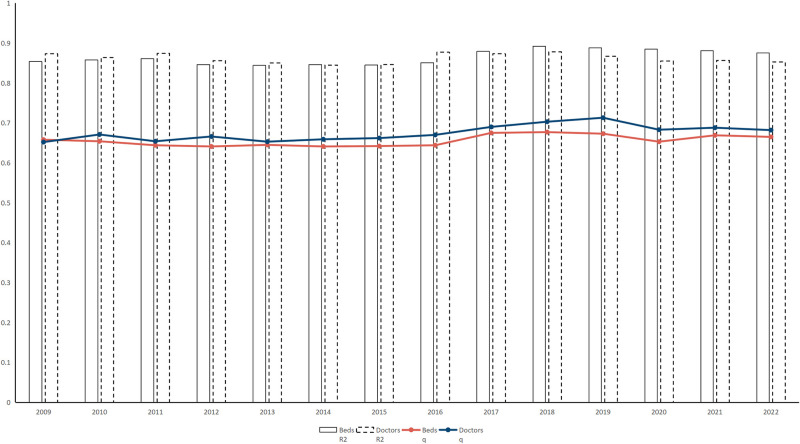
The Zipf’s law confidence of Health resources in the YRD Region, 2009–2022. Notes: Parameter *q* represents the scale factor for the number of beds and doctors.

This analysis employed Eq (2) to model the number of beds and physicians in each city in the YRD region, correlating them with their respective rankings, using Stata 17: Fig 4 illustrates the dimensions of Zipf’s law and the goodness-of-fit for these models from 2009 to 2022. Remarkably, the goodness-of-fit scores for the models pertaining to both beds and physicians consistently exceeded 0.8, indicating strong correlations. Additionally, these models attained statistical significance at the 1% level, emphasizing the effective depiction of the relationship between health resources (beds and physicians) and urban economic size within the YRD region according to Zipf’s law [54].

Between 2009 and 2022, the Zipf’s law coefficients for bed size and the number of physicians in the YRD cities were consistently below 1. This suggests that top-ranked cities, such as Shanghai, Hangzhou, and Hefei do not monopolize the distribution of healthcare resources, indicating a relatively balanced allocation across the entire YRD urban agglomeration. Furthermore, Zipf’s law coefficients for bed size and number of physicians demonstrated a comparable developmental trend, exhibiting an upward trajectory with 2019 as the turning point. From 2009 to 2019, the coefficients for beds and physicians increased from 0.658 to 0.673 and from 0.652 to 0.713, respectively. Subsequently, from 2019 to 2022, these coefficients declined slightly for beds (0.673–0.665) and physicians (0.713–0.682). This shift can be attributed to the COVID-19 pandemic, which has disrupted the development of medical resources in YRD cities, leading to an intensified focus on healthcare systems. Consequently, there has been significant growth in healthcare resources, accompanied by a reduction in disparities among cities. Moreover, the Zipf’s law coefficient for physicians is notably higher than that for beds, indicating that larger cities are more attractive to physicians, leading to faster growth in the number of physicians in these areas compared to smaller cities. This trend reinforces the concentration of healthcare resources in larger urban centers, with the distribution of physicians among cities gradually shifting towards a standard "Zipf’s law" distribution [55].

### Spatial and temporal evolution of the allometric scaling of urban economic and health resources

#### Vertical allometric analysis

By employing Formula (4), separate allometric scaling models for the number of beds, physicians, and the urban economy were developed. These models underwent double logarithmic linear fitting, yielding tailored models that effectively captured the allometric scaling dynamics of beds, physicians, and urban economies in the YRD region.

Table 3 reveals that the goodness-of-fit for both the allometric scaling models of bed and physician numbers in relation to the urban economy in the YRD region from 2009 to 2022 consistently exceeded 0.74, indicating a generally robust fit. Furthermore, throughout this period, the longitudinal allometric growth ratings for both city economy bed size and physician size consistently maintained positive levels of 2. This classification signifies that the growth rates in the number of beds and physicians in the region outpaced those of the urban economy. Additionally, the coefficient depicting the relationship between the urban economy and the number of physicians exhibits a fluctuating downward trend from 2009 to 2022, decreasing gradually from 1.2162 to 1.1723. Concurrently, the scale coefficient, reflecting the ratio of the urban economy to the number of physicians, demonstrated an annual decrease, moving from 1.3127 in 2009 to 1.1666 in 2022. This trend indicates a relative decrease in the growth rate of physicians compared to that of the urban economy.

**Table 3:** Estimated results of vertical allometric scaling of urban and health resources.

Year	Beds				Doctors			
	*b*	*cons*	*R2*	Allometric level	*b*	*cons*	*R2*	Allometric level
2009	1.2162	4.86	0.7516	Positive 2	1.3127	4.73	0.8413	Positive 2
2010	1.2199	4.94	0.7599	Positive 2	1.2029	5.83	0.7724	Positive 2
2011	1.2122	5.09	0.7547	Positive 2	1.2873	5.21	0.8644	Positive 2
2012	1.2038	5.15	0.7583	Positive 2	1.2386	5.65	0.858	Positive 2
2013	1.188	0.31	0.7571	Positive 2	1.2406	5.62	0.8416	Positive 2
2014	1.2048	5.14	0.7644	Positive 2	1.2301	5.75	0.845	Positive 2
2015	1.1936	5.23	0.7484	Positive 2	1.2173	5.86	0.8259	Positive 2
2016	1.1941	5.24	0.7419	Positive 2	1.2504	5.55	0.8554	Positive 2
2017	1.1958	5.45	0.7929	Positive 2	1.2065	6.01	0.8492	Positive 2
2018	1.1973	5.45	0.7848	Positive 2	1.1911	6.16	0.8521	Positive 2
2019	1.2035	5.43	0.8244	Positive 2	1.1619	6.47	0.8851	Positive 2
2020	1.2200	5.21	0.8	Positive 2	1.1867	6.16	0.8574	Positive 2
2021	1.1918	5.61	0.7982	Positive 2	1.1764	6.32	0.8458	Positive 2
2022	1.1723	5.81	0.773	Positive 2	1.1666	6.42	0.828	Positive 2

Notes: Beds refers to the allometric model of the city’s economy-bed resource; The term "doctors" refers to the allometric model relating a city’s economy to its doctors’ resources, where *b* represents the allometric scaling index.

### Horizontal allometric analysis of urban economy and beds

(1) Horizontal allometric analysis of the urban economy-bed size relationship

**Fig 5:**
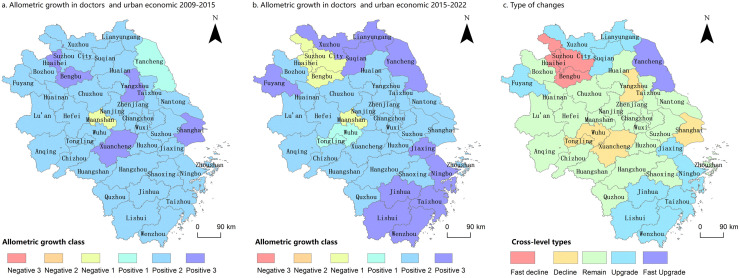
Allometric of beds number allometric types and their changes in YRD Cities, 2009–2022. Notes: Based on the standard map GS (2019)1822 of the standard map service website of the Ministry of Natural Resources, the map boundary was not modified during ArcGIS production; a fast decline in the graph refers to a decrease in the allometric class of two or more grades during the study period, and a fast upgrade refers to an increase in the allometric class of two or more grades during the study period.

Spatial changes in the horizontal allometric relationship of bed resources from 2009 to 2022 were visualized using the ArcGIS software. This visualization employed the horizontal allometric relationship between bed resources and the city economy for each city in the YRD region, spanning 2009–2015 and 2016–2022, as shown in Fig 5.

As illustrated in Fig 5A, from 2009 to 2015, the YRD region exhibited predominantly positive allometric growth in city economy and bed numbers. Positive allometric cities were classified into primary, secondary, and tertiary levels. In total, 40 cities exhibited positive allometric growth, accounting for 97.56%. Of these, 34 cities (82.92%) were classified as secondary positive allometrics, and five cities (12.19%) were classified as tertiary positive allometrics. Ma’anshan was the only city that exhibited negative allometric growth. As illustrated in Fig 5B, from 2016 to 2022, the predominant growth type shifts to a secondary positive allometry. This category included 37 cities, with two identified as primary positive allometric and 24 as secondary positive allometric cities. Additionally, 11 tertiary iso-speed cities were primarily located in southern Zhejiang and northern Jiangsu provinces. Four cities (9.75%) exhibited negative allometric growth and were classified as secondary negative allometric growth cities: Bengbu, Huaibei, Ma’anshan, and Suzhou.

As illustrated in Fig 5C, the trend analysis of anisotropy changes during the study period revealed that 19 cities (46.34% of the total) experienced changes in their allometric classification. Among these, 11 cities exhibited upward changes, primarily in southern Zhejiang Province and northern Jiangsu Province, reflecting a faster growth in bed size relative to economic development. Conversely, eight cities exhibited downward changes, primarily in central Anhui Province and the northern parts of the region, suggesting slower growth in bed size relative to economic development.

(2) Horizontal allometric analysis of urban economy and doctors

**Fig 6:**
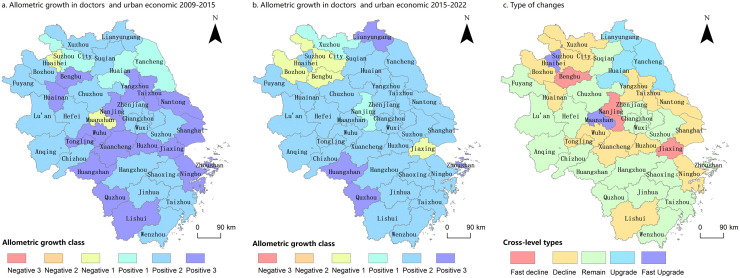
Allometric of doctors, number of allometric types and their changes in YRD Cities, 2009–2022. Notes: Based on the standard map GS (2019)1822 of the standard map service website of the Ministry of Natural Resources, the map boundary was not modified during ArcGIS production; a fast decline in the graph refers to a decrease in the allometric class of two or more grades during the study period, and a fast upgrade refers to an increase in the allometric class of two or more grades during the study period.

The spatial variations in doctors’ horizontal allometric relationships from 2009 to 2022 were analyzed using ArcGIS software through horizontal allometric analysis of doctors’ resources and city economies across each city in the YRD region for 2009–2015 and 2016–2022 (as illustrated in Fig 6).

As illustrated in Fig 6A between 2009 and 2015, cities in the YRD region predominantly exhibited positive economic-doctor-number-scale allometric scaling. Specifically, 39 cities (95.12% of the total) exhibited this characteristic. These cities were geographically widespread. Among them, 19 cities (46.34%) primarily located in the central and northern Jiangsu Province, southern Zhejiang Province, and central Anhui Province exhibited second-level positive allometric scaling. Additionally, 17 cities (41.46%) exhibited tertiary-level positive allometric characteristics distributed in a ring-like pattern around the regions exhibiting positive second-level allometric scaling. Only two cities, Huaibei and Maanshan exhibited negative allometric scaling. As illustrated in Fig 6B, the predominant trend in allometric scaling remained positive between 2016 and 2022. Specifically, 37 cities exhibited positive growth in allometric scaling. This included two cities at the first level, 31 cities at the second level covering most of the YRD, and four cities at the third level: Huangshan, Lianyungang, Quzhou, and Zhoushan. Four cities–Bengbu, Bozhou, Jiaxing, and Suzhou–exhibited negative allometric scaling, accounting for 4.88% of the total.

As illustrated in Fig 6C, the analysis of the changes in anisotropy types during the study period revealed significant trends. Of the 41 cities, 22 (53.66%) exhibited shifts in allometric type. Among these, five cities primarily located in the northern part of Jiangsu Province, exhibited upward shifts, indicating faster relative growth in the number of doctors compared with the speed of economic development. Conversely, 17 cities concentrated in a ring-shaped distribution around the southern part of Jiangsu Province and northern part of Anhui Province exhibited downward shifts, indicating a decline in the relative growth rate of doctors.

## Discussion

### The impact of changes in the size structure of health resources in the YRD region

Economic development is the primary driver of health-resource growth [56]. Changes in the size of the urban economy and health resources in the YRD region manifest primarily in three aspects: (1) There is a significant centralization trend in the urban economy and health resources, although the distribution of medical resources remains more balanced. (2) The distribution structure of health resources transitioned from an equalization phase to a more standardized " rank-scale distribution. Small- and medium-sized cities are driving the rapid growth of health resources in the YRD region. (3) The centralization of physician resources occurred more rapidly than that of bed resources.

Specifically, cities in the YRD centered around provincial capitals such as Shanghai, Nanjing, Hangzhou, and Hefei have undergone rapid economic growth, leading to a pronounced economic disparity between central and peripheral cities. Despite the centralization trends, the structure of health resources in the YRD, particularly in central cities such as Shanghai, Hangzhou, and Nanjing, remains relatively balanced and does not deviate significantly from the norm, contrary to the findings of Jiang et al. [57]. This is because China typically employs a population-based allocation model for health resources, resulting in smaller disparities in health resources between cities in the YRD than in urban economies. Consequently, the polycentric structure of urban health resources in the YRD reflects the combined influence of the economy, city administrative hierarchy, and population dynamics.

Narrowing the health-resource gap is a primary objective of China’s health system reform, with numerous policies implemented to redistribute health resources from advantaged to disadvantaged cities to reduce disparities in health-resource allocation. However, this study reveals that the trend towards health-resource centralization remains unmitigated, with increasing Zipf coefficients for both bed and physician resources approaching one, which indicates a growing concentration in larger cities and a shift towards a hierarchical scale distribution. This phenomenon is likely driven by economic centralization, which catalyzes the convergence of the population and resources toward economically advantaged cities [58]. In these cities, economic prosperity not only supports an increase in health resources but also meets the growing demands driven by population expansion. This trend mirrors developments in education [59] and pension resources [60] across China. Additionally, the COVID-19 pandemic temporarily disrupted this centralization trend, impacting the region-wide distribution of health resources. Nonetheless, the predominant trajectory of the hierarchical scale distribution persists, propelled by the economic allure and policy focus of the provincial capital and major urban centers. Furthermore, the concentration of physician resources exceeds that of bed resources, which is attributable to the superior economic incentives and living conditions that economically advantaged cities offer physicians, thus intensifying the inflow and further concentrating these resources.

### Implications of changes in allometric growth for the YRD Region

Changes in the structure of urban health resources and the economy are driving shifts in the patterns of allometric growth across most YRD regions. Changes in the allometric growth relationship between urban health resources and the economy in the YRD region are primarily reflected in three key aspects. (1) The growth rate of urban health resources in the YRD exceeds that of the urban economy. (2) The relative growth rate of health resources followed a downward trajectory. (3) The growth of health resources in the YRD cities is characterized by diffusion.

Specifically, during the study period, the longitudinal anisotropic growth ratio of the economy to the number of beds and doctors in YRD cities exceeded one, indicating that the relative growth rates of both beds and doctors surpassed the economic growth rate, which can be attributed to the gradual transition of the YRD economy from rapid to medium- to high-speed growth over the past decade. Although the healthcare industry in China developed later, the rapid transformation of health concepts and increasing demand for health services have driven the government to increase its investment in the sector. Consequently, health resources remain in a phase of relatively high-speed growth, and the mismatch in their development compared to the regional economy has led to an allometric growth relationship between the two, which has established an allometric growth relationship between health resources and the economy. This also suggests that current health resources in the YRD region are yet to meet the growing demand for health services driven by economic development. Additionally, the decreasing allometric growth coefficients for beds and physicians during the study period may be linked to changes in the developmental stages of China’s healthcare industry [61]. Xue et al. [62] concluded that China’s health system reform since 2009 could be divided into three phases: infrastructure expansion, deepening reforms, and high-quality development. The transition between these phases influenced changes in the allometric growth coefficient of beds and doctors, shifting the focus from quantitative expansion to prioritizing quality growth. Moreover, while Chen et al. argued that the growth of health resources in the region primarily stems from the rapid growth of central cities [63], this study found that growth originated predominantly in small- and medium-sized cities. Specifically, small and medium-sized cities in the YRD exhibited a higher degree of allometric growth, whereas large cities demonstrated a relatively steady rate.

### Policy insights

Both the size structure of health resources and allometric growth results in the YRD suggest that health-resource development is dependent on regional economic growth. Although rapid economic growth has accelerated centralization in major cities, this trend is inevitable, and mirrors patterns observed in developed countries such as Japan and Korea. Given that centralization in the YRD is an inevitable outcome of economic development, the rational allocation of health resources within urban agglomerations require attention to both the evolving scale and structure of these resources and their alignment with each city’s economic development. According to Zipf, the urban system distribution becomes more effective as its q-value approaches 1. Therefore, reasonably controlling the scale structure of health resources can effectively prevent overconcentration in large cities and promote balanced development across small and medium-sized cities in the YRD [64]. Consequently, the YRD should leverage its Regional Integration and Development Plan to establish a coordinated regional decision-making mechanism that reflects its collective interests and long-term YRD goals. This mechanism optimizes resource allocation throughout the region through a robust and unified framework. Initially, the integration of the regional economy and health system should be designed to align with ongoing economic and health-resource development trends in the YRD. Enhancing the hierarchical scaling of regional central cities is crucial to maximize their catalytic roles. Concurrently, it is essential to avoid disorderly competition among cities regarding resource allocation. In particular, when integrating the YRD, it is important to focus on the complementarity and differences in economic and medical resources among regions and promote a multi-tiered city-scale system that fosters gradient development among large, medium, and small cities to achieve optimality.

Furthermore, with the integrated development of the YRD region elevated to a national strategy, the allometric growth coefficient of urban health resources in the YRD gradually decreased, marking a shift from rapid expansion to a focus on coordinated and high-quality development aligned with the city’s economic growth. At this juncture, coordinating the relationship between economic development and health-resource allocation is crucial. Given the government’s enhanced macro-control, health-resource allocation must align with regional economic demands while concurrently improving the quality and efficiency of these allocations. This entails not only increasing health-resource inputs and the proportion of healthcare expenditure in public finance but also advancing healthcare system reforms [65].

### Potential limitations and prospects

This study has several limitations. First, owing to data collection challenges, this study focused exclusively on the YRD region. Given the economic disparities across China, these findings may not fully represent the national characteristics of healthcare resource distribution. Moreover, this analysis is confined to the urban scale, examining the scale structure of urban health resources and the allometric growth relationship between the economic scale and health resources. Theoretically, data at smaller scales, such as the county level, may yield conclusions that are closer to reality and provide a more precise depiction of the relationship between health resources and economic scale [66]. Thus, future research should be extended to broader geographical areas and more granular scales to enhance the accuracy of the findings.

Second, although this study analyzed the temporal evolution and spatial distribution of its findings, it did not examine the mechanisms driving the allometric growth relationship between urban health resources and the economy. In future studies, we plan to employ various statistical models, including geodetectors, artificial neural networks, and ordinary least squares regression to explore the formation mechanisms of allometric growth and regional variability. This approach aims to offer more nuanced insights into the coordinated development of urban health resources and the economy.

## Conclusions

This study analyzed the economic scale and health resources (doctors/beds) of 41 cities in the YRD region from 2009 to 2022. It examined the spatial and temporal evolution and developmental patterns of the scale structure of health resources as well as the allometric growth relationship between these factors. Initially, the scale coefficients of bed and physician resources were calculated using the law of city size to analyze the trends in health-resource development within the region. An urban health-resource economy allometric growth model was developed to explore the intrinsic relationship between health resources and economic growth. This study found a trend of centralization in the structure of urban health resources in YRD, with both bed and physician resource-scale factors showing an upward trend. Notably, physician resources demonstrated a significant increase of 9.35% over the previous 13 years, compared to a 2.28% increase in bed resources. In addition, the relationship between health resources and the economy was uniformly and positively allometric, indicating that health resources grew faster than the economy. Moreover, the coefficient of allometric growth in the YRD showed a decreasing trend from 2009 to 2022, with a decrease of 3.61% for bed resources and 11.13% for physician resources.

1. The economic and health resources of the YRD cities exhibit a spatial distribution characterized by being "high in the east and low in the west, high in the south and low in the north." This pattern is accompanied by growing advantages in the central cities of the region in terms of economic and health resources, reflecting a spatial pattern of "one pole and many nuclei." Both the numbers of doctors and beds in the YRD region showed a trend toward relative equalization. In terms of development trends, physicians and bed sizes are transitioning towards standardized "rank-size distributions," and these changes in the profile of urban health resources are influencing shifts in the allometric growth types across much of the YRD, laying the foundation for further investigation into its growth dynamics and evolution.
2. In the YRD cities, the allometric growth of the economy in relation to physician and bed sizes exhibited a weak pattern of health expansion, where the relative growth rates of bed and physician sizes were slightly higher than those of the urban economy. Overall, the scalar indices for economy-bed and doctor sizes exhibit variable declines from 2009 to 2022. From the perspective of city scale, the scaling index for small and medium-sized cities exceeds that of large cities. Most small and medium-sized cities have shifted from weak to strong patterns of health expansion. This suggests that the rapid growth of health resources in smaller cities significantly affected the evolution of health-scale dynamics across the YRD region.

## Supporting information

S1 Table Data. (XLSX)
