# Correction: Spatial-temporal evolution of the allometric relationship between urban economic and health resources in the Yangtze River Delta urban agglomeration

**DOI:** 10.1371/journal.pone.0322414

**Published:** 2025-04-07

**Authors:** Jing Deng, Qianwen Song, Huan Liu, Zicheng Jiang, Xingxing He, Chengzhi Ge, Dexun Li

The first and last name of the first author are incorrectly switched. The correct name is: Jing Deng. The correct citation is: Deng J, Song Q, Liu H, Jiang Z, He X, Ge C, et al. (2025) Spatial-temporal evolution of the allometric relationship between urban economic and health resources in the Yangtze River Delta urban agglomeration. PLoS ONE 20(1): e0314315. https://doi.org/10.1371/journal.pone.0314315.

## References

[1] Jing D, Song Q, Liu H, Jiang Z, He X, Ge C, et al. Spatial-temporal evolution of the allometric relationship between urban economic and health resources in the Yangtze River Delta urban agglomeration. PLoS One. 2025;20(1): e0314315. doi: 10.1371/journal.pone.0314315 39854516 PMC11759377

